# Correlation Between Tumor Response and Survival Outcomes in Patients with Advanced Gastric Cancer Receiving Ramucirumab and Paclitaxel as Second-Line Therapy

**DOI:** 10.1007/s12029-022-00865-5

**Published:** 2022-09-15

**Authors:** Giandomenico Roviello, Catalano Martina, Costanza Winchler, Irene De Gennaro Aquino, Francesca Papa, Eleonora Buttitta, Gemma Rossi, Lorenzo Antonuzzo

**Affiliations:** 1 Department of Health Sciences, Section of Clinical Pharmacology and Oncology, University of Florence, Viale Pieraccini, 6, 50139 Florence, Italy; 2 School of Human Health Sciences, University of Florence, Largo Brambilla 3, 50134 Florence, Italy; 3 Department of Experimental and Clinical Medicine, University of Florence, 50134 Florence, Italy; 4 Medical Oncology Unit, Careggi University Hospital, 50134 Florence, Italy

**Keywords:** Ramucirumab, Paclitaxel, Gastric cancer, Tumor response, Overall survival, Progression free survival

## Abstract

**Background:**

Gastric cancer (GC) is one of the leading causes of cancer-related death worldwide. The first-line treatment for GC is a combination of platinum and fluoropyrimidine-based therapy. Based on the positive results of RAINBOW and REGARD trials, ramucirumab either alone or in combination with paclitaxel has proved to be a safe and active option for second-line treatment in GC patients.

**Material and methods:**

Advanced GC patients who received a 28-day cycles of ramucirumab and paclitaxel until disease progression or unacceptable toxicity were evaluated. Eligible patients had ECOG PS ≤ 1 and adequate organ function. Baseline characteristics were assessed for progression-free survival (PFS) and overall survival (OS). The Kaplan–Meier method and Cox proportional-hazards regression models were used for survival analyses.

**Results:**

In our single institution experience, we included a total of 67 patients. A median OS of 8 months and a median PFS of 4 months, were recorded. In patients experiencing an initial partial response (PR), we observed a significant association between tumor response and survival outcomes (OS and PFS). The OS and PFS were 15 and 11 months in patients who experienced PR compared to 8 and 4 months in patients without PR (*p* = 0.02; *p* = 0.04).

**Conclusion:**

Treatment with ramucirumab plus paclitaxel yielded the highest overall response rate reported to date for patients with previously treated advanced GC. In our experience, the initial tumor response is associated with a greater survival benefit which could be further improved by the identification of biomarkers predicting response.

**Supplementary Information:**

The online version contains supplementary material available at 10.1007/s12029-022-00865-5.

## Introduction

Gastric cancer (GC) is the fifth most common cancer and the third leading cause of cancer death worldwide [1]. The high mortality rate in GC is mainly due to the lack of specific early manifestations, subsequently leading to the late diagnosis and treatment [2, 3].

For patients with advanced or recurrent GC combination chemotherapy regimens consisting of fluoropyrimidines and platinum, with trastuzumab, in HER2 positive cancer, or a third agent such as taxane or anthracycline are the gold standard in the first line treatment [4–8]. However, the prognosis for these patients remains poor, with a median overall survival (OS) that does not exceed 10–12 months [9, 10]. Furthermore, the majority of patients do not respond or relapse within a short time after the end of first-line therapy and only about one-third of patients receive second-line chemotherapy treatments [11]. Various cytotoxic agents both as monotherapy and in combination have been extensively studied in the second-line setting with minimal benefit in these patients [12, 13].

However, although clinical studies have shown that second-line therapy improves OS compared to BSC to a statistically significant extent in actual clinical practice, second-line regimen may not be offered to all patients, mainly due to poor PS experienced after first-line therapy [14–16]. Furthermore, in translating the survival benefit from clinical trials to real life, regional ethnic differences must be considered. In fact, almost all Asian patients with metastatic GC receive second-line therapy while in Western countries, less than half of patients receive progressive second-line treatment after first-line therapy [17].

More recently, the use of ramucirumab, a human monoclonal antibody (IgG1) vascular endothelial growth factor receptor-2 (VEGFR-2) antagonist, plus paclitaxel versus paclitaxel alone in second line showed to increase median OS (9.6 months vs 7.4 months) and progression free survival (PSF) (4.4 months vs 2.9 months) in all subgroups of the phase III (RAINBOW) trial [18]. Ramucirumab plus paclitaxel demonstrated the highest objective response rate (ORR) of 28% reported in the second line setting compared with 16% in the paclitaxel alone group (*p* = 0.0001). Ramucirumab alone versus best supportive care (BSC) confirmed to improve OS in the REGARD study [19]. Based on these findings, paclitaxel plus ramucirumab became the standard second-line treatment for advanced GC. In this study, we retrospectively evaluated the efficacy of ramucirumab plus paclitaxel in patients with advanced GC, presenting, details of the association between partial tumor response and survival outcomes.

## Material and Methods

### Patients’ Population

We examined the medical records of patients with metastatic GC who received a second-line therapy with ramucirumab and paclitaxel in a single institution from January 2016 to April 2021.

Eligibility criteria for treatment with ramucirumab and paclitaxel included the following: presence of histologically confirmed metastatic gastric cancer; Eastern Cooperative Oncology Group Performance Status (ECOG PS) of ≤ 2; adequate bone marrow, renal and liver function (including, absolute neutrophil count ≥ 1.5 × 10^9^L, platelet count ≥ 100,000/mm^3^, and hemoglobin level ≥ 9 g/dl; estimate glomerular filtrate rate ≥ 60 ml/min bilirubin level ≤ the upper limit of normal). The exceptions related to enzymatic alterations (e.g., Gilbert Syndrome) or values of Hb < 9 g/dl from gastric bleeding, were considered individually. Patients received previous first-line treatment for metastatic disease and at least one course of therapy with ramucirumab plus paclitaxel have been considered for our study.

#### Treatment Schedule

Patients received a 28-day cycle of ramucirumab (8 mg/kg intravenously) administered on days 1 and 15 and paclitaxel (80 mg/m^2^ intravenously) on days 1, 8, and 15. The treatment was continued until disease progression or unacceptable toxicity. The dose reduction of drugs at starting the treatment was agreed depending on ECOG PS, comorbidities or toxicities from previous treatments. Dose modification and interruption of treatment were performed in relation to the criteria established in the pivotal clinical studies [18].

### Response Evaluation and Statistical Analysis

Computed tomography scans were performed every 12 weeks or before is clinically indicated. Tumor response was assessed in accordance with RECIST 1.1 [20]. PFS was defined as the interval from the start of treatment with ramucirumab and paclitaxel to the evidence of progressive disease (PD) or death from any cause. Overall survival represents the duration of patient survival from the time of treatment initiation. The best overall response is the best response recorded from initiation of treatment up to disease progression/relapse and may be complete response (CR), partial response (PR), stable disease (SD) or PD. For this analysis, patients have been dichotomized in two groups: patients that achieved PR and those that do not achieved PR (SD and/or PD). The Kaplan–Meier method and Cox proportional hazards regression models were used for survival analyses. Hazard ratio (HR) together with 95% confidence interval (CI) were provided for Cox proportional hazards regression analyses. A two-sided *p*-value < 0.05 was considered statistically significant. Statistical analyses were performed using STATA v.2012. This study was approved by the Comitato Etico Regionale for clinical experimentation of Toscana region (Italy) Area Vasta Centro section, number: 14912_oss.

## Results

### Patient’s Characteristics

A total of 67 patients with metastatic GC treated with ramucirumab and paclitaxel between January 2016 and April 2021 were included in our single institution experience. The mean age was 66 years (range, 33–80) with 32.8% more than 70 years; ECOG-PS was 1 in 34 (50.7%) of patients. All patients had previously received a treatment with platinum and fluoropyrimidine-based chemotherapy regimens, and 40 patients (59.7%) had received previous surgery. Thirty-nine (58.2%) patients experienced time to disease on first-line therapy < 6 months. The number of metastatic sites was ≥ 3 in 21 (31.3%) patients and peritoneal involvement was equally present in 21 (31.3%) patients. Patient characteristics are shown in Table 1.

**Table 1:** Patient’s characteristics

	**All patients** **(N = 67)**		**Subgroup patients**	
		**PR** **7 (10.4%)**	**SD + PD** **60 (89.5%)**	***P***
**Age, years** Median (range) ≥ 70	66 (33–80) 22 (32.8%)	68 (49–75) 3 (42.9%)	66 (33–80) 19 (31.7%)	0.9 0.4
**Sex** Male	47 (70.1%)	4 (57.1%)	43 (71.7%)	0.3
**ECOG PS** 1	34 (50.7%)	3 (42.9%)	31 (51.2%)	0.4
**Tumor location** Stomach	48 (71.6%)	7 (100%)	41 (68.3%)	0.1
**Number of metastatic sites** ≥ 3	21 (31.3%)	2 (28.6%)	19 (31.7%)	0.6
**Previous Surgery** Yes	40 (59.7%)	4 (57.1%)	36 (60%)	0.6
**Time to progressive disease on first-line therapy** < 6 months	9 (58.2%)	6 (85.7%)	33 (55%)	0.1
**Peritoneal metastases** Yes	21 (31.3%)	1 (14.3%)	20 (33.3%)	0.3

*N* Number, *ECOG PS *Eastern Cooperative Oncology Group Performance Status, *PR* partial response, *SD *stable disease, *PD *progression disease, *P p* value

## Treatment Results

The median cycles received were 4 (range, 1–24). The number of patients who required a reduced dose of ramucirumab or paclitaxel was 11 (16.4%) and 45 (67.2%), respectively. Treatment was equally delayed for the two drugs. Twenty-six patients discontinued ramucirumab and 29 (43.3%) patients discontinued paclitaxel due to disease progression, toxicity, or other reasons. Thirty-three (49.2%) patients received a subsequent line of chemotherapy (Table 2). A median OS of 8 months (range, 7–10) and a median PFS of 4 months (range, 3–5) were recorded (Table 3).

**Table 2:** Dose reduction, treatment delay and treatment interruption

	**All patients** **(N = 67)**		**Subgroup patients**	
		**PR** **7 (10.4%)**	**SD + PD** **60 (89.5%)**	***P***
**Cycles** Median (range)	4 (1–24)	10 (5–22)	4 (1–24)	**0.01**
**Dose reduction** **(ramucirumab)**	11 (16.4%)	1 (14.3%)	10 (16.7%)	0.7
**Treatment delay** **(ramucirumab)**	26 (38.8%)	5 (71.4%)	21 (35%)	0.1
**Treatment interruption** **(ramucirumab)**	26 (38.8%)	2 (28.6%)	24 (40%)	0.4
**Dose reduction** **(paclitaxel)**	45 (67.2%)	3 (42.9%)	42 (70%)	0.4
**Treatment delay** **(paclitaxel)**	26 (38.8%)	5 (71.4%)	21 (35%)	0.1
**Treatment interruption** **(paclitaxel)**	29 (43.3%)	3 (42.9%)	26 (43.3%)	0.6
**Subsequent** **line of therapy**	33 (49.2%)	7 (100%)	26 (43.3%)	** < 0.01**

*N* number*, PR* partial response*, SD* stable disease*, PD *progression disease, *P* *p *value

**Table 3:** Best response, PFS and OS according to score population

	**All patients** **(N = 67)**		**Subgroup patients**	
		**PR** **7 (10.4%)**	**SD + PD** **60 (89.5%)**	***P***
**PFS** months (95% CI)	4 (3–5)	11 (6–23)	4 (3–5)	0.02/ 0.42 (0.19–0.94)
**OS** months **(**95% CI)	8 (7–10)	15 (7–28)	8 (7–9)	0.04/ 0.43 (0.18–0.98)

*N* number*, PFS* progression free survival, *OS* overall survival*, PR* partial response*, SD* stable disease, *PD* progression disease

### Correlation Between Response Tumor and Survival Parameters

**Fig. 1:**
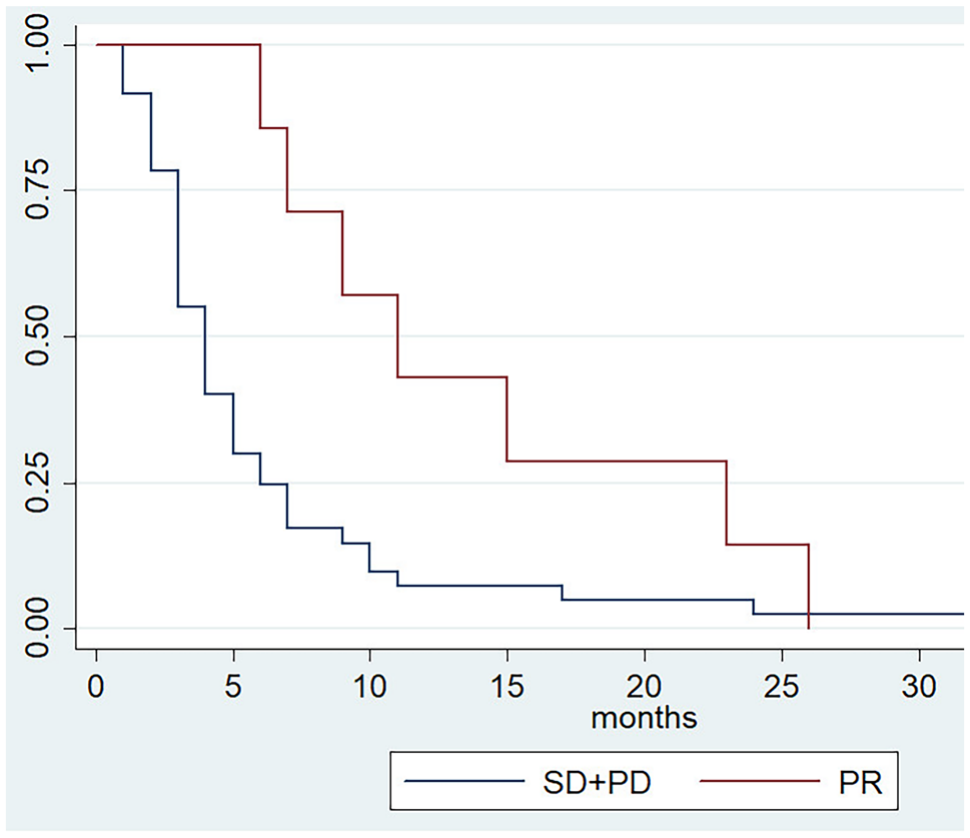
Progression free survival according to tumor response

**Fig. 2:**
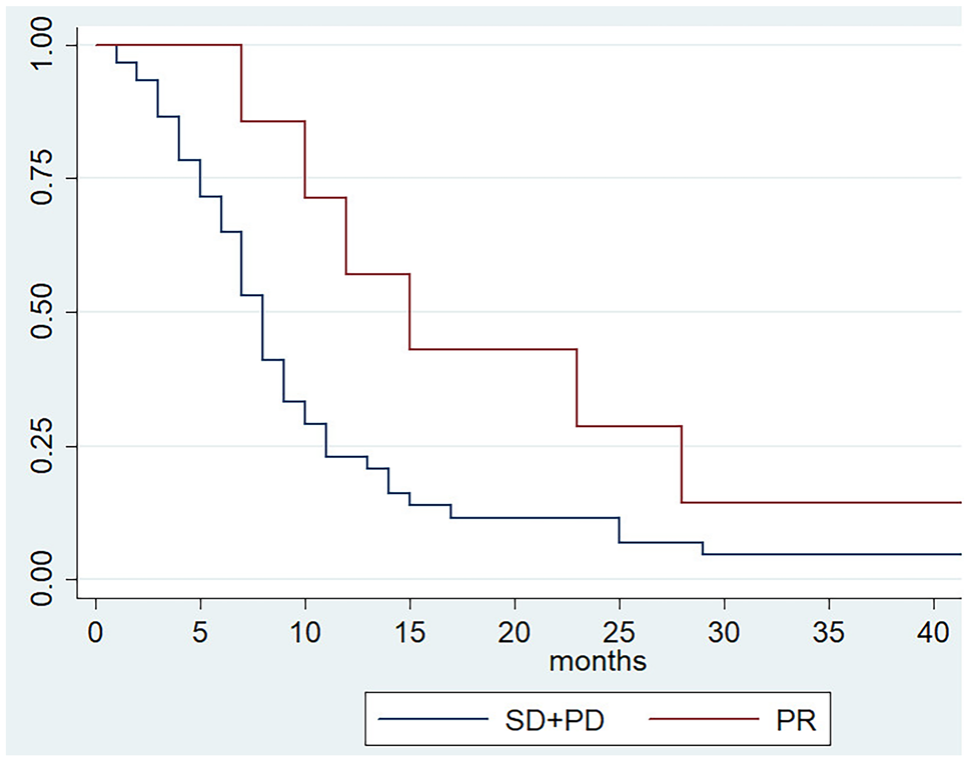
Overall survival according to tumor response

We focused the analysis on patients with clinical benefit that is PR (no CRs were observed) at least at the first disease reassessment. Clinical and treatment characteristics of patients who experienced PR (N = 7) and of those who have not reached PR (SD + PD, N = 60) are shown in Table 1. Patients in PR group received a median of 10 [5–22] cycles of chemotherapy compared to 4 [1–24] cycles in patients without PR (*p* = 0.01). No statistical differences were observed concerning dose reduction, treatment delayed and treatment interruption between the two groups; all patients with PR received a subsequent line of chemotherapy, contrary to 43.3% of patients without PR (Table 2). In this study, we explored the association of survival outcomes with PR observing a significant correlation with both OS and PSF. Specifically, PR was associated with better OS (HR 0.42, 95% CI 0.19–0.94; *p* = 0.02) and PFS (HR 0.43, 95% CI 0.18–0.98; *p* = 0.04) compared to non-PR (Figs. 1 and 2). To allow for better interpretation of the data, characteristic of patients experienced PD at first tumor reassessment were separately reported (Table 1 Supplementary information).

#### Univariate and Multivariate Analysis

Cox regression analysis has been performed on the entire cohort of 67 patients to assess the associations between clinical-pathological variables of interest and better survival outcomes. Risk variables assessed included age, gender, tumor location, number of metastatic sites, previous surgery, ECOG PS, peritoneal metastases, time to progressive disease on first-line therapy and partial response.

In the univariate analysis, ECOG PS = 1 (versus 0) (HR, 1.44; 95% CI, 1.05–1.93; *p* = 0.02), presence of peritoneal metastases (HR, 1.83; 95% CI, 1.10–2.35; *p* = 0.03), time to progressive disease on first-line therapy < 6 months (HR, 1.44; 95% CI, 1.21–1.80; *p* = 0.03) and previous surgery (HR, 1.10; 95% CI, 1.05–1.77; *p* = 0.04) were correlated with worse survival, whereas PR (HR, 0.43; 95% CI, 0.18–0.98; *p* = 0.04) has been correlated with better survival outcomes.

Multivariate analysis confirmed ECOG PS (HR, 1.24; 95% CI, 1.01–1.76; *p* = 0.04) and peritoneal metastases (HR, 2.03; 95% CI, 0.403.68; *p* < 0.01) as negative prognostic factors for survival and PR (HR, 0.55; 95% CI, 0.44–0.927; *p* = 0.04) as prognostic positive variable.

## Discussion

A large part of patients with GC are initially diagnosed with unresectable or metastatic disease, and first-line chemotherapy guarantees a median overall survival often not exceeding 12 months [21, 22].

To date, the RAINBOW trial demonstrated the highest second-line response rate in patients with advanced gastric or gastroesophageal junction (GEJ) adenocarcinoma. Significant improvements in PFS (HR 0.635; 95%CI 0.536, 0.752; *p* < 0.0001), OS (HR 0.807; 95% CI 0.678–0.962; *p* = 0.0169), and overall response rates (27.9% *p* = 0.0001) have been reached [18]. Moreover, a subgroup analysis in Western population, showed a median OS of 8.5 months for ramucirumab and paclitaxel and 5.9 months for paclitaxel alone. Median PFS was 4.2 and 2.8 months in the two group, respectively with an ORR of 26.8% in the combination arm (*p* = 0.0004) [23]. Subsequently, real-life studies on ramucirumab and paclitaxel as second-line therapy in metastatic GC have achieved results in terms of OS and PSF overlapping [24, 25].

In our single institution study, median OS [8 months, range 7–10] and PFS [4 months, range 3–5] were in line with the pivotal trial. However, focusing on patients who achieved PR to treatment, a define benefit was recorded in terms of survival. In these patients, compared with patients that do not experienced PR, OS and PFS were 15 months (HR 0.43; 95% CI 0.18–0.98; *p* = 0.04) and 11 months (HR 0.42; 95% CI 0.19–0.94; *p* = 0.02), respectively (Figs. 1 and 2). At the multivariate analysis, we identified ECOG PS = 1 (HR, 1.24; 95% CI, 1.01–1.76; *p* = 0.04) and presence of peritoneal metastases (HR, 2.03; 95% CI, 0.403.68; *p* < 0.01) as factors correlated with worse survival outcomes, whereas PR (HR, 0.55; 95% CI, 0.44–0.927; *p* = 0.04) has been correlated with higher survival. Thus, although second-line treatments have guaranteed an improvement in terms of survival, the prognosis of these patients remains poor and only a few benefit from it. This emphasizes the need for identifying predictive biomarkers to better select patient and direct it to second-line chemotherapy with ramucirumab and paclitaxel or clinical trials.

Previously, depth of response (DpR) has been correlated with post progression survival in subgroups of gastric cancer patients receiving second-line chemotherapy, indicating DpR as possible new predictor for efficiency [26]. However, the predictive value of DpR is not sure, which may be related to other factors, such us the mutation status of Human Epidermal Growth Factor Receptor 2 and treatment methods, among others.

A recent prospective study has suggested the prognostic value of some circulating factors such as neutrophil-to-lymphocyte ratio (NLR) and myeloid-derived suppressor cells (MDSCs) on survival outcomes in GC patients receiving ramucirumab plus paclitaxel treatment [27]. Another study suggested that the occurrence of high-grade neutropenia can predict response to treatment with ramucirumab and paclitaxel. In this analysis, patients who experienced grade ≥ 3 neutropenia had a PFS of 6.6 months (95% CI 3.3–8.4) and an OS of 11 months (95% CI 5.9–13.1) compared to 4.4 months (95% CI 3.9–5.2) and 8.7 months (95% CI 7.8–10.1) for patients with lower grade neutropenia [28].

Natsume et al. identified a correlation between aberrant expression of placental growth factor (PlGF) and ramucirumab responders and non-responders. OS (*p* = 0.046) and PFS (*p* = 0.016) were significantly shorter in the PlGF-high group than in the PlGF-low group. Overall response rates were 50% and 0% in the PlGF-low and high group, respectively [29].

However, despite the various efforts made, no predictive biomarkers have yet been identified and the mechanism underlying the response or resistance to the combination of ramucirumab and paclitaxel remains unclear [30].

Moreover, Cascinu et al. reported the correlation between tumor response, and the symptom palliation in the intent to treat population of the RAINBOW study, as also observed in patients with metastatic breast cancer receiving chemotherapy [31].

This study presents several limitations mainly due to the retrospective nature of the data collection, the limited number of patients included, and a single Oncologic Center involved. Moreover, the inclusion of patients with primary progressive disease, who have an extremely poor survival, in the subgroup which do not experience PR, amplifies the prognostic impact of PR itself. However, while aware that tumor response should be associated with improved survival, this may not necessarily occur [32]. What we want to underline with this work is the statistically significant difference in OS and PFS that we observed between the patients who have achieved PR and who have not achieved PR, which further pushes us to continue looking for biomarkers capable of selecting patients guaranteeing the best therapeutic choice Figs. 1 and 2.

## Conclusion

Second-line treatment with ramucirumab plus paclitaxel is the currently recognized standard of care for patients with advanced gastric adenocarcinoma or GEJ previously treated with recommended first-line therapy. Although with many limitations, we have reported statistically significant survival benefits in patients who exhibit a partial response as the best response to treatment. Since the prognosis of these patients remains very limited, the identification of predictive biomarkers of response could improve the selection of patients who benefit most from this association.

### Supplementary Information

Below is the link to the electronic supplementary material.

Supplementary file1 (DOCX 19 KB)

## Data Availability

The data used to support the findings of this study are available from the corresponding author upon request.
