# Supplementary material for: Correlation Between Tumor Response and Survival Outcomes in Patients with Advanced Gastric Cancer Receiving Ramucirumab and Paclitaxel as Second-Line Therapy
Source: J Gastrointest Cancer. 2022 Sep 15;54(3):802–8. doi: 10.1007/s12029-022-00865-5 (PMC10613139; doi:10.1007/s12029-022-00865-5)
Supplement: Supplementary file 1 — Supplementary file1 (DOCX 19 KB) [file 12029_2022_865_MOESM1_ESM.docx]

**Table 1 Sup:** Patients with progression disease characteristics

| Progression Disease  N=39 (58.2%) | |
| --- | --- |
| Age, years  Median (range)  ≥70 | 64 (33-80)  10 (25.6%) |
| Sex  Male | 27 (69.2%) |
| ECOG PS  1 | 21 (53.8%) |
| Tumor location  Stomach | 25 (64.1%) |
| Number of  metastatic sites  ≥3 | 14 (35.9%) |
| Previous Surgery  Yes | 21 (53.8%) |
| Time to progressive disease  on first-line therapy  <6 months | 21 (53.8%) |
| Peritoneal metastases  Yes | 13 (33.3%) |

Number (N); Eastern Cooperative Oncology Group Performance Status (ECOG PS).
